# Intracolonial genetic variation affects reproductive skew and colony productivity during colony foundation in a parthenogenetic termite

**DOI:** 10.1186/s12862-014-0177-0

**Published:** 2014-08-14

**Authors:** Satoshi Miyazaki, Miho Yoshimura, Ryota Saiki, Yoshinobu Hayashi, Osamu Kitade, Kazuki Tsuji, Kiyoto Maekawa

**Affiliations:** 1 Graduate School of Science and Engineering, University of Toyama, Gofuku, Toyama, 930-8555, Japan; 2 Department of Hygiene and Public Health, Tokyo Women’s Medical University, Shinjuku-ku, 162-8666, Tokyo, Japan; 3 Graduate School of Environmental Science, Hokkaido University, Sapporo, 060-0810, Hokkaido, Japan; 4 College of Science, Ibaraki University, Mito, 310-8512, Ibaraki, Japan; 5 Faculty of Agriculture, University of the Ryukyus, Nishihara, 903-0213, Okinawa, Japan

**Keywords:** Intracolonial genetic variation, Colony efficiency, Reproductive skew, Eusociality, Parthenogenesis, Caste differentiation, Nymph, Reticulitermes speratus

## Abstract

**Background:**

In insect societies, intracolonial genetic variation is predicted to affect both colony efficiency and reproductive skew. However, because the effects of genetic variation on these two colony characteristics have been tested independently, it remains unclear whether they are affected by genetic variation independently or in a related manner. Here we test the effect of genetic variation on colony efficiency and reproductive skew in a rhinotermitid termite, *Reticulitermes speratus*, a species in which female-female pairs can facultatively found colonies. We established colonies using two types of female-female pairs: colonies founded by sisters (i.e., sister-pair colonies) and those founded by females from different colonies (i.e., unrelated-pair colonies). Colony growth and reproductive skew were then compared between the two types of incipient colonies.

**Results:**

At 15 months after colony foundation, unrelated-pair colonies were larger than sister-pair colonies, although the caste ratio between workers and nymphs, which were alternatively differentiated from young larvae, did not differ significantly. Microsatellite DNA analyses of both founders and their parthenogenetically produced offspring indicated that, in both sister-pair and unrelated-pair colonies, there was no significant skew in the production of eggs, larvae, workers and soldiers. Nymph production, however, was significantly more skewed in the sister-pair colonies than in unrelated-pair colonies. Because nymphs can develop into winged adults (alates) or nymphoid reproductives, they have a higher chance of direct reproduction than workers in this species.

**Conclusions:**

Our results support the idea that higher genetic variation among colony members could provide an increase in colony productivity, as shown in hymenopteran social insects. Moreover, this study suggests that low genetic variation (high relatedness) between founding females increases reproductive skew via one female preferentially channeling her relatives along the reproductive track. This study thus demonstrated that, in social insects, intracolonial genetic variation can simultaneously affect both colony efficiency and reproductive skew.

## Background

Recent evolutionary theories predict that genetic variation among group members can affect characteristics of social insect colonies in two ways. First, an increase in genetic variation among workers can enhance colony efficiency by facilitating division of labor [1] and increased disease resistance [2],[3]. Second, increased genetic variation among colony members can affect intracolonial conflicts, often by intensifying them [4]. Although there is empirical evidence supporting the above views, to our knowledge, no study has tested the two effects simultaneously. Thus, it remains unknown whether genetic variation has a direct impact on both characteristics. A study testing these two effects would provide us an opportunity to understand how intracolonial genetic variation affects evolution and maintenance of colony characteristics.

We focused on colony founding in the rhinotermitid termite *Reticulitermes speratus*. In this species, as in other termites, newly emerged winged adults (alates) generally form a male–female pair, which founds a colony together and reproduces sexually [5]. However, Matsuura and Nishida [6] reported that occasionally when two female alates failed to find males, they could co-found a colony and reproduce parthenogenetically. This homosexual cooperation benefits both females, because their survival probability is markedly enhanced when compared to females singly founding a colony [7],[8]. Given the limited dispersal (flight) ability of alate termites in this species, relatedness of such co-foundress naturally varies, depending on the distribution of colonies in a population. This termite is therefore suitable for testing the two possible predicted effects of intracolonial genetic variation.

Reproductive skew, the degree of uneven partitioning of reproductive output among group members, is a colony characteristic that can reflect intracolonial conflicts. Transactional models of reproductive skew, in which group members yield reproductive shares to each other in return for cooperation, predict that reproductive skew becomes larger as relatedness increases [9]. Empirical tests of this and other predictions of reproductive skew models are often problematic [4],[10], however, because predictions are affected by assumptions and by several parameters other than relatedness (i.e., group size, fighting ability, ecological effect on independent reproduction, etc., [11]-[13]). One such parameter that affects the outcome is group size. Many reproductive skew models assume that a group consists of two individuals (cooperating) or one individual (not-cooperating). However, the group size of real animals is often more than two individuals and can vary, giving rise to a statistical problem in defining reproductive skew [14]. Colony founding by *R. speratus* is advantageous in testing reproductive skew models, because the group size is either two (pair founding) or one (individual or haplometrotic founding), thus meeting a fundamental assumption of basic reproductive skew models [15].

**Figure 1:**
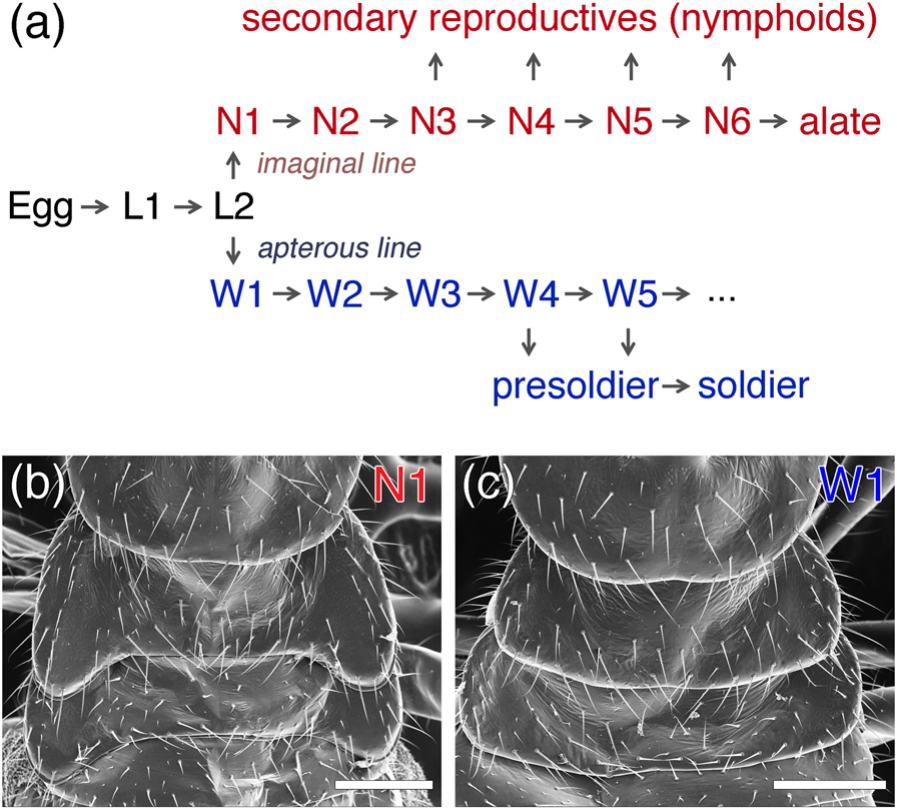
**Caste developmental pathway of*Reticulitermes speratus*(a), and the thorax of N1 (b) and W1 (c).** L, N and W indicate larva, nymph and worker, respectively. Note that N1 has wing buds on meso- and metanotum. Scale bars indicate 0.2 mm.

The genetic factors associated with determining caste trajectory into nymphs vs. workers have been identified in this species; parthenogenetically produced eggs are genetically predisposed to develop into nymphs. However, a percentage of eggs can develop into workers in response to environmental stimuli, e.g., the presence of reproductive individuals [16]. Nymphs can molt into secondary reproductives (nymphoids; Figure 1a), and nymphoids parthenogenetically produced by the primary queen become the main reproductive force in natural colonies of this species [17]. Thus, the production of nymphs can be an index of reproductive output in colonies. Based on the above evolutionary theories, we tested our prediction that a reproductive skew in nymph production is affected by genetic relatedness of co-foundresses, and that genetic variation among sterile workers positively affects colony efficiency.

To test whether intracolonial genetic variation affects colony efficiency and reproductive skew in *R. speratus*, we studied two types of incipient female-female colonies under laboratory conditions: colonies founded by closely related individuals (i.e., sister-pair colonies) and those founded by non-kin (i.e., unrelated-pair colonies). Colonies were censused and compared at 15 months after colony foundation, and the genotypes of individual colony members were determined using microsatellite DNA.

## Results

### Rapid colony development in unrelated-pair colonies

**Figure 2:**
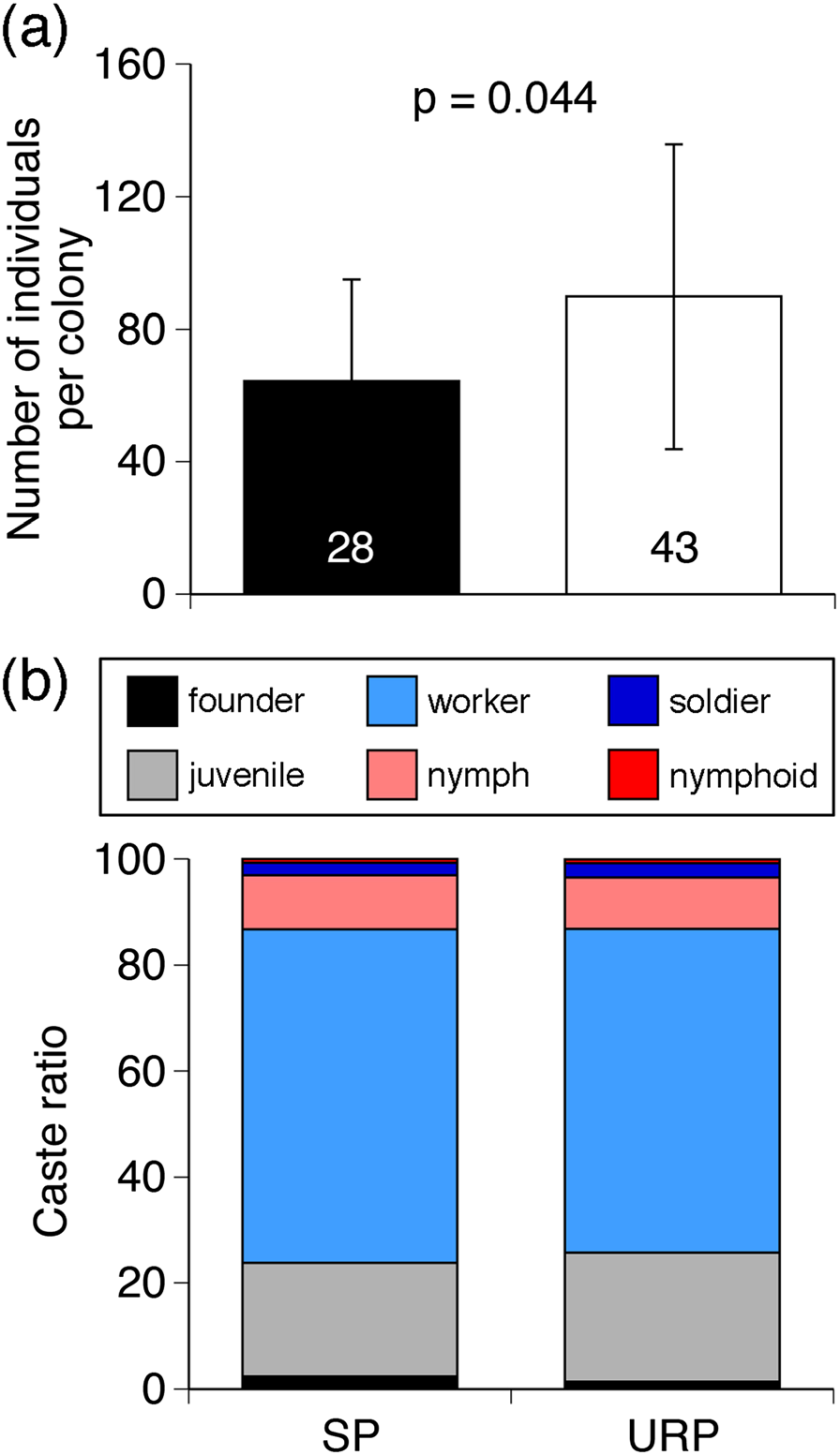
**Effects of relatedness between founding females on the colony growth and caste ratio. (a)** The colony size (means ± S.D. values) at 15 months after colony establishment. The sizes were significantly different between colony types (generalized linear mixed model (GLMM) analysis, p = 0.044, Table 1). **(b)** Caste ratio between colony types in each colony at 15 months after colony establishment. The nymph/worker ratio did not significantly differ between colony types (GLMM analysis, p = 0.430, Table 1). Numerical values in the bar indicate numbers of colonies examined. SP and URP indicate sister-pair and unrelated-pair colonies, respectively.

At 15 months after colony foundation, 78.6 and 64.0% of founding females survived in 28 sister-pair colonies and 43 unrelated-pair colonies, respectively. The survival rates did not differ significantly between these two colony types (generalized linear mixed model (GLMM), Table 1). A GLMM analysis of the effects of colony characters including colony type (sister-pair or unrelated-pair) on colony size (the sum of numbers of queens, eggs, larvae, workers, soldiers, nymphs and nymphoids) revealed that the colony size was significantly affected by colony type, but neither the founders’ survival rates nor the colony type × survival rate interaction (Table 1). Specifically, colony size in unrelated-pair colonies was significantly larger than those in the sister-pair colonies (Figure 2a). Nymph/worker ratio, however, was not affected by colony type (Figure 2b), colony size, number of surviving queens, nor any interactions among these factors (GLMM, Table 1), indicating that intracolonial genetic variation between founders did not affect the nymph-worker caste ratio at the colony level. The number of neither nymphoids nor soldiers differed between colony types (GLMM, Table 1).

**Table 1:** Effects of social characters on colony efficiency (the number of termites)

**Response variable**	**Explanatory variable**	**Z value**	**P value**
Founders’ survival rate	colony type (CT)	1.835	0.067
Colony size	colony type (CT)	−2.017	0.044*
	founders’ survival rate (FSR)	−1.048	0.295
	interaction between CT & FSR	1.094	0.274
Nymph/worker ratio	colony type (CT)	−0.790	0.430
	founders’ survival rate (FSR)	−0.533	0.594
	colony size (CS)	0.306	0.759
	interaction between CT & FSR	1.149	0.251
	interaction between CT & CS	1.265	0.206
	interaction between FSR & CS	0.280	0.779
	interaction between CT, FSR & CS	−1.433	0.152
Number of soldiers	colony type (CT)	0.405	0.685
	founders’ survival rate (FSR)	0.421	0.674
	colony size (CS)	−0.060	0.952
	interaction between CT & FSR	−0.181	0.856
	interaction between CT & CS	−0.872	0.383
	interaction between FSR & CS	0.010	0.992
	interaction between CT, FSR & CS	0.374	0.708
Number of nymphoids	colony type (CT)	0.165	0.869
	founders’ survival rate (FSR)	−0.725	0.469
	colony size (CS)	−0.623	0.533
	interaction between CT & FSR	−0.216	0.829
	interaction between CT & CS	0.119	0.905
	interaction between FSR & CS	−0.029	0.977
	interaction between CT, FSR & CS	−0.084	0.933

Social characters include colony types (i.e., sister-pair and unrelated-pair colonies), founders’ survival rate, colony size and their interactions. “*” indicates P < 0.05 in generalized linear mixed model (GLMM) analyses.

### Nymph production skewed toward one of the paired founding females in sister-pair colonies

**Figure 3:**
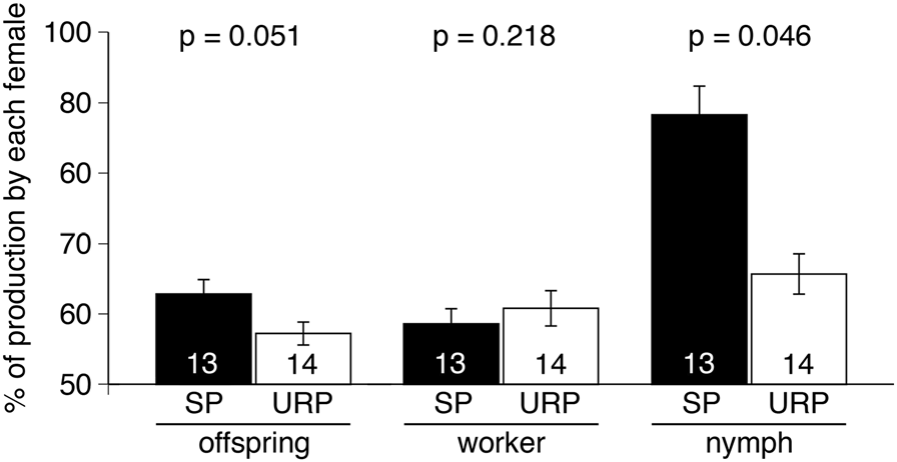
**Proportions of offspring (including all individuals except for queens), workers, and nymphs produced by one of paired founding females.** The proportions (means ± S.D. values) were compared between sister-pair (SP, black) and unrelated-pair (URP, white) colonies using GLMM analyses. Numerical values in the bar indicate numbers of colonies examined.

Genetic differences (shown as different microsatellite alleles) between the paired founding females were observed (or estimated from worker genotypes, in cases of colonies with only 1 female surviving; see Methods) in 13 sister-pair and 14 unrelated-pair colonies. In these colonies, microsatellite DNA analyses were performed to assign parentage to offspring, which included juveniles (eggs and larvae), workers, soldiers, nymphs and nymphoids (Additional file 1 and Additional file 2); the higher proportion (≧50%) between two founding females was then defined as the skew of offspring production. The skew of nymph production was significantly affected by colony type, both directly and indirectly, while the skew of juvenile-, worker-, soldier-, and offspring-production were not (Table 2, GLMM). Colony size and survival rate of founding females did not significantly affect skews (Table 2, GLMM). These results indicate that nymph production was more skewed in sister-pair colonies than in unrelated-pair colonies (Figure 3, Additional file 3).

**Table 2:** Effects of social characters on reproductive skew

**Response variable**	**Explanatory variable**	**Z value**	**P value**
Skew of offspring production	colony type (CT)	1.952	0.051
	founders’ survival rate (FSR)	1.558	0.119
	colony size (CS)	1.141	0.254
	interaction between CT & FSR	−1.738	0.082
	interaction between CT & CS	−1.585	0.113
	interaction between FSR & CS	−1.534	0.125
	interaction between CT, FSR & CS	1.493	0.136
Skew of juvenile production	colony type (CT)	1.482	0.138
	founders’ survival rate (FSR)	0.422	0.673
	colony size (CS)	0.301	0.763
	interaction between CT & FSR	−1.336	0.182
	interaction between CT & CS	−1.365	0.172
	interaction between FSR & CS	−0.342	0.733
	interaction between CT, FSR & CS	1.283	0.199
Skew of worker production	colony type (CT)	1.232	0.218
	founders’ survival rate (FSR)	1.131	0.258
	colony size (CS)	1.232	0.218
	interaction between CT & FSR	−1.434	0.152
	interaction between CT & CS	−1.315	0.189
	interaction between FSR & CS	−1.581	0.114
	interaction between CT, FSR & CS	1.502	0.133
Skew of nymph production	colony type (CT)	1.997	0.046*
	founders’ survival rate (FSR)	−0.230	0.818
	colony size (CS)	0.175	0.861
	interaction between CT & FSR	−1.197	0.231
	interaction between CT & CS	−2.664	0.008**
	interaction between FSR & CS	0.049	0.961
	interaction between CT, FSR & CS	2.152	0.031*
Skew of soldier production	colony type (CT)	−0.001	0.999
	founders’ survival rate (FSR)	0.452	0.651
	colony size (CS)	0.287	0.774
	interaction between CT & FSR	0.001	0.999
	interaction between CT & CS	0	1.000
	interaction between FSR & CS	−0.211	0.833
	interaction between CT, FSR & CS	0	1.000

For the explanatory values, see Table 1. “*” and “**” indicates P < 0.05 and 0.01 in GLMM analyses, respectively. Skew of nymphoids could not be analyzed because of small numbers of colonies producing nymphoids (3 of 13 sister-pair colonies and 3 of 14 unrelated-pair ones, see Additional files 1, 2 and 3).

## Discussion

In this study, we showed that genetic variation among colony members affected colony characteristics in *R. speratus* from two perspectives. First, in unrelated-pair colonies, colony growth was higher. Second, in sister-pair colonies, nymph production was skewed toward one of paired females. These results demonstrate that intracolonial genetic variation had the two distinct effects on colony characteristics: a positive effect on colony efficiency, and a negative effect on reproductive monopoly, i.e., reproductive skew.

### Colony growth increased in unrelated-pair colonies with higher genetic variation among workers

In the honeybee *Apis mellifera*, intracolonial genetic variation promotes both task specialization and resistance to diseases, resulting in increases in colony productivity [1]. Similar relationships between genetic variation and colony productivity were observed in ants [18]-[20] and a wasp [21], taxa whose socialities evolved independently of the honeybee’s [22],[23]. Our results showed that, in *R. speratus*, colony size was larger in unrelated-pair colonies (i.e., with higher intracolonial genetic variation), than in sister-pair colonies (Figure 2a). Furthermore, in both sister-pair and unrelated-pair colonies, worker production was only slightly skewed toward one founding female (Figure 3), suggesting that there is an advantage to increasing genetic variation among workers. In a termite society, then, the increase in worker genetic variation promotes colony productivity, probably through effective division of labor and resistance to diseases, as in social hymenopterans.

According to the Red Queen Hypothesis for the evolution of sexual reproduction, genetic variation generated by sexual recombination among host individuals prevents pathogens from adapting to particular host genotypes [24]-[26]. In social insects intracolonial genetic variation among workers is thought to decrease intracolonial transmission of genotype-specific pathogens [27],[28], and effective division of labor, particularly hygienic behavior such as corpse removal and colony cleaning, can prevent the spread of infectious diseases in insect colonies [29]. Intracolonial genetic variation is known to promote task specialization among workers [18],[30]-[32], suggesting that genetic variation can improve disease resistance through division of labor. In social hymenopterans, worker genetic variation can be increased not only by sexual recombination but also by polyandry and/or polygyny [1]. In the basal termite *Zootermopsis angusticollis*, outbred colonies were more resistant to fungal and microbial pathogens than inbred colonies, indicating that genetic variation arising from outbreeding increased disease resistance through social interactions such as trophallaxis and grooming [33],[34]. Because *R. speratus* at the colony founding stage faces high risk of infection by bacteria, fungi and natural pathogens [7], increased levels of genetic variation are expected to have an impact on survivorship during colony foundation. Thus, worker genetic variation stemming from pleometrosis between unrelated females may promote disease resistance.

### Reproductive skew in nymph production

In *R. speratus*, the production of nymphs is a key step in colony reproduction, because nymphs can molt into alates or differentiate into nymphoid reproductives when primary reproductives senesce or die. Our results indicate that a skew in nymph production was positively correlated with relatedness of the individuals in female pairs (Figure 3); this supports one of the transactional models of reproductive skew (the concession model) in which a dominant individual controls the subordinate’s fraction of total group reproduction [9]. According to the concession model, reproductive skew may be further increased under strong ecological constraints, when it would be preferable for the subordinate to stay in the colony rather than to leave the colony and reproduce alone [35]. It has been demonstrated that, in *R. speratus*, a female that founds a colony alone exhibits twice the mortality of a female in a founding pair, even under experimental conditions [6]. Although to date there have been few studies supporting these predictions of concession models [4], the current study strongly supports them. Skew models incorporate many parameters (e.g., relatedness, group size, fighting ability, ecological harshness on independent reproduction), of which total experimental control in order to fit to the assumptions is often less feasible. Our results supporting concession models might be due to our system, which more closely fits the models’ assumptions (e.g. two cooperative individuals) than other systems. The current study additionally sheds light on a new aspect. There was a statistically significant colony growth × relatedness interaction effect on reproductive skew (Table 2), suggesting that these are not independent and thus should be studied simultaneously. Future theoretical studies should focus on the possibility that if an enhanced colony’s resource level is due to cooperation of unrelated females rather than related females, it can in turn increase the skew or vice versa (Additional file 4). Those are new facets of reproductive skew theory.

Parthenogenetically produced offspring are genetically predisposed to develop into nymphs in *R. speratus*[16]. However, in the presence of nymphoid reproductives, nymphal development is inhibited in one quarter of the offspring; they instead become workers [16]. In the present study, c.a. 80 percent of the offspring developed into workers in incipient female-female colonies (Figure 2b). This suggests that interaction with reproductive individuals may inhibit nymphal development. The present study further demonstrated that nymph production was influenced by the relatedness between founding females, whereas the production of workers was not (Figure 3, Additional file 4). These results suggest that skewed nymph production in these termites is controlled via nepotistic regulation of nymphal development by reproductives, in which some offspring parthenogenetically produced by a founding female are released from the induction of worker development. Although the proximate mechanism of this regulation remains unclear, physical contact and pheromonal regulation are possibilities.

## Conclusions

Our study clearly revealed that, in incipient colonies founded by two females of *R. speratus*, increased intracolonial genetic variation had two distinct impacts on social characteristics. First, it improved colony growth while maintaining the nymph/worker caste ratio, probably through effective division of labor and resistance to diseases. Second, it decreased reproductive skew via nepotistic regulation of nymphal development. To our knowledge, this is the first report testing and demonstrating these effects of intracolonial genetic variation simultaneously. Simultaneous testing such as this can serve to link colony characteristics in insect societies with the evolutionary significance of intracolonial genetic variation.

## Methods

### Termite colonies

Twelve mature termite colonies were collected in Toyama and Ishikawa Prefectures, Japan, in April 2008–2010 (colony no. A to L). Log sections housing the termites were brought back to the laboratory and maintained in plastic boxes in constant darkness.

### Female-female colony establishment

After the emergence of alates (winged adults) from these colonies, the sexes of individuals were determined using the morphology of abdominal tergites [36]. Adults that had shed their wings were chosen randomly and female-female pairs were established (see [37]) with individuals from the same colony (using 4 colonies, total 28 sister-pairs) and from two different colonies (using 10 colonies, total 43 unrelated-pairs). Each pair was placed in a 20 mL glass vial with c.a. 8 g of mixed sawdust food (Mitani, Ibaraki, Japan) and kept at 25°C in constant darkness. At 15 months after colony foundation, 1802 and 3862 termites from 28 sister-pair and 43 unrelated-pair colonies, respectively, were sampled and stored in 99.5% ethanol. After determining their developmental stages and castes, 614 and 746 termites from the sister-pair and the unrelated-pair colonies were used for genotyping (see “Genotype analysis”).

### DNA extraction and amplification

Total DNA was extracted from the entire insect (larvae and eggs), a leg (queens) or the head (other castes), using the DNeasy Tissue Kit (Qiagen, Japan) for queens, and 10% Chelex 100 (Bio-Rad, USA) for other individuals. Five microsatellite loci (Rf6-1, Rf21-1, Rf24-2, Rs02, and Rs03) were used for this study. Primer sequences for the amplification of Rf- and Rs-loci are given in Vargo [38] and Hayashi et al. [39], respectively.

### Genotype analysis

First, genotypes of founding queens in each colony were analyzed using the five microsatellite loci. Three unrelated-pair colonies without surviving queens were excluded from this analysis. For 12 sister-pair colonies and 24 unrelated-pair colonies that contained only 1 surviving queen and offspring, we inferred the genotype of the dead queen from data of the existing queen and workers. If we could not obtain evidence that a dead queen produced eggs or larvae, we excluded these colonies from analysis; in such cases a queen had died at least 1 month prior to sampling. Based on the queens’ genotyping data, 13 sister-pair colonies and 14 unrelated-pair colonies, in which genotypes of both founding queens were distinguished, were used for this analysis. Then, PCR products of 614 and 746 termites from the sister-pair and the unrelated-pair colonies were electrophoresed on 7% polyacrylamide gels (7 h, 100 V and 15 mA), and stained with an ethidium bromide solution (10 mg/ml). Amplified DNA fragments were observed using Dolphin-Doc (Kurabo, Japan), and genotyping was performed with the use of Dolphin-1D software (Kurabo).

### Statistical analyses

For evaluating how intracolonial genetic diversity affects social characteristics in *R. speratus* colonies, generalized linear mixed model (GLMM) analyses were conducted. Fixed effects were colony type (i.e., relatedness between founding queens), founders’ survival rate, colony size, and their interactions, while founded colony and founders’ source colony were included as random effects. Numbers of termites were analyzed using GLMMs with Poisson error distribution and log link function, whereas data for reproductive skew (number of offspring produced by more productive or less productive founders) and caste ratio were analyzed with binomial error distribution and logit link function. Significance of partial regression coefficients was evaluated by Wald tests. P values of less than 0.05 were considered significant. These analyses were conducted using R ver. 3.1.0 (available at http://cran.r-project.org/) and the lme4 package.

## Competing interests

The authors declare that they have no competing interests.

## Authors’ contributions

SM and MY performed genetic analyses and drafted the manuscript. RS carried out the ecological experiments and prepared Figures 1, 2 and 3. YH and OK analyzed molecular data. KT participated in the design of the study and performed the statistical analysis. KM conceived of the study, and participated in its design and coordination and helped to draft the manuscript. All authors read and approved the final manuscript.

## Additional files

## Supplementary Material

Additional file 1 Inferred numbers of offspring produced by each female in sister-pair colonies. Click here for file

Additional file 2 Inferred numbers of offspring produced by each female in unrelated-pair colonies. Click here for file

Additional file 3 **Proportions of juveniles (including eggs and larvae), soldiers, and nymphoids produced by one of paired founding females.** The proportions (means ± S.D. values) were compared between sister-pair (SP, black) and unrelated-pair (URP, white) colonies using GLMM analyses. Numerical values in the bar indicate numbers of colonies examined. Click here for file

Additional file 4 **Skew of nymph production affected by the interactions among colony type, colony size and founders’ survival rate.** In unrelated-pair colonies, colony size increased the skew of nymph production, regardless of the number of founding females (narrow solid and broken lines). In sister-pair colonies with both founding females, colony size increased the skew (thick solid line). In sister-pair colonies where only one of two founding females survived, colony size decreased the skewed nymph production (thick broken line). Here, the interactions between colony type and colony size, and also interactions among these two and founders’ survival rate significantly affected the skew of nymph production (see Table 2). Click here for file
