# Supplementary material for: Intracolonial genetic variation affects reproductive skew and colony productivity during colony foundation in a parthenogenetic termite
Source: BMC Evol Biol. 2014 Aug 14;14:177. doi: 10.1186/s12862-014-0177-0 (PMC4236541; doi:10.1186/s12862-014-0177-0)
Supplement: Additional file 1 — Inferred numbers of offspring produced by each female in sister-pair colonies. [file s12862-014-0177-0-S1.pdf]

Additional file 1. Inferred numbers of offspring produced by each female in sister-pair colonies

| Colony | Queen | Egg and larva | Worker | Soldier | Nymph | Nymphoid | Sum of offspring |
|---|---|---|---|---|---|---|---|
| AA-1 | F1 | 2 | 13 | 1 | 8 | 0 | 24 |
| | F2 | 2 | 13 | 0 | 0 | 0 | 15 |
| | Sum[*1] | 4/4 | 26/63 | 1/1 | 8/8 | 0/0 | 39/76 |
| AA-2 | F1 | 1 | 15 | 1 | 7 | 0 | 24 |
| | F2 | 1 | 14 | 0 | 0 | 0 | 15 |
| | Sum[*1] | 2/10 | 29/29 | 1/1 | 7/7 | 0/0 | 39/47 |
| AA-3 | F1 | 6 | 19 | 3 | 10 | 2 | 40 |
| | F2[*2] | 3 | 5 | 0 | 2 | 0 | 10 |
| | Sum[*1] | 9/10 | 24/35 | 3/3 | 12/12 | 2/2 | 50/62 |
| AA-4 | F1 | 6 | 16 | 1 | 6 | 0 | 29 |
| | F2 | 2 | 11 | 0 | 1 | 0 | 14 |
| | Sum[*1] | 8/9 | 27/29 | 1/1 | 7/7 | 0/0 | 43/46 |
| AA-5 | F1 | 0 | 18 | 0 | 7 | 0 | 25 |
| | F2 | 1 | 12 | 0 | 1 | 0 | 14 |
| | Sum[*1] | 1/1 | 30/67 | 0/0 | 8/8 | 0/1 | 39/77 |
| AA-6 | F1 | 7 | 14 | 1 | 8 | 0 | 30 |
| | F2 | 6 | 7 | 0 | 1 | 0 | 14 |
| | Sum[*1] | 13/16 | 21/39 | 1/1 | 9/9 | 0/0 | 44/65 |
| AA-7 | F1 | 13 | 17 | 1 | 3 | 0 | 34 |
| | F2[*2] | 6 | 14 | 1 | 4 | 0 | 25 |
| | Sum[*1] | 19/31 | 31/40 | 2/2 | 7/7 | 0/0 | 59/80 |
| AA-8 | F1 | 4 | 8 | 2 | 12 | 0 | 26 |
| | F2 | 7 | 10 | 0 | 1 | 0 | 18 |
| | Sum[*1] | 11/14 | 18/82 | 2/2 | 13/13 | 0/0 | 44/111 |
| AA-9 | F1 | 11 | 12 | 0 | 0 | 0 | 23 |
| | F2 | 6 | 8 | 1 | 6 | 0 | 21 |
| | Sum[*1] | 17/27 | 20/50 | 1/1 | 6/6 | 0/0 | 44/84 |
| AA-10 | F1 | 0 | 20 | 1 | 8 | 0 | 29 |
| | F2[*2] | 5 | 15 | 0 | 0 | 0 | 20 |
| | Sum[*1] | 5/5 | 35/35 | 1/1 | 8/8 | 0/0 | 49/49 |
| BB-11 | F1 | 0 | 12 | 2 | 16 | 0 | 30 |
| | F2[*3] | 1 | 9 | 1 | 1 | 0 | 12 |
| | Sum[*1] | 1/1 | 21/26 | 3/3 | 17/17 | 0/0 | 42/47 |
| KK-12 | F1 | 10 | 15 | 2 | 10 | 1 | 38 |
| | F2[*2] | 6 | 15 | 0 | 7 | 0 | 28 |
| | Sum[*1] | 16/23 | 30/52 | 2/2 | 17/17 | 1/1 | 66/95 |
| KK-13 | F1 | 8 | 18 | 2 | 4 | 0 | 32 |
| | F2 | 12 | 12 | 0 | 0 | 0 | 24 |
| | Sum[*1] | 20/86 | 30/56 | 2/2 | 4/4 | 0/0 | 56/148 |

[*1]Numbers examined/existing numbers in the colony

[*2]Dead queen, existence was inferred from the genotype analysis of workers
