# Supplementary material for: Intracolonial genetic variation affects reproductive skew and colony productivity during colony foundation in a parthenogenetic termite
Source: BMC Evol Biol. 2014 Aug 14;14:177. doi: 10.1186/s12862-014-0177-0 (PMC4236541; doi:10.1186/s12862-014-0177-0)
Supplement: Additional file 2 — Inferred numbers of offspring produced by each female in unrelated-pair colonies. [file s12862-014-0177-0-S2.pdf]

Additional file 2. Inferred numbers of offspring produced by each female in unrelated-pair colonies

Colony	Queen	Egg and larva	Worker	Soldier	Nymph	Nymphoid	Sum of offspring
CF-1	F1	11	14	3	4	0	32
	F2[*2]	8	11	1	1	0	21
	Sum[*2]	19/43	25/103	4/4	5/5	0/0	53/155
CF-2	F1[*2]	7	21	1	7	0	36
	F2	11	13	3	6	0	33
	Sum[*1]	18/18	34/80	4/4	13/13	0/0	69/115
DF-3	F1[*2]	9	13	2	6	0	30
	F2	15	9	1	2	0	27
	Sum[*1]	24/96	22/107	3/3	8/8	0/0	57/214
DF-4	F1	8	19	1	3	0	31
	F2[*2]	5	15	1	7	0	28
	Sum[*1]	13/13	34/54	2/2	10/10	0/0	59/79
EF-5	F1	9	16	1	7	0	33
	F2[*2]	14	8	2	3	0	27
	Sum[*1]	23/72	24/103	3/3	10/10	0/0	60/188
EF-6	F1[*2]	9	14	1	18	0	42
	F2	6	11	2	13	0	32
	Sum[*1]	15/91	25/55	3/3	31/36	0/0	74/185
EF-7	F1	0	22	3	2	0	27
	F2	0	14	0	1	0	15
	Sum[*1]	0/0	36/40	3/3	3/3	0/0	42/46
EF-8	F1	12	16	3	2	0	33
	F2[*2]	9	7	0	14	0	30
	Sum[*1]	21/63	23/97	3/3	16/16	0/0	63/179
EF-9	F1	1	14	2	11	0	28
	F2	4	14	0	6	0	24
	Sum[*1]	5/21	28/113	2/2	17/17	0/0	52/153
GI-10	F1	0	8	2	9	1	20
	F2	0	4	1	9	0	14
	Sum[*1]	0/18	12/62	3/3	18/18	1/1	34/102
GI-11	F1	3	15	1	4	0	23
	F2	1	12	1	3	0	17
	Sum[*1]	4/9	27/30	2/2	7/7	0/0	40/48
GI-12	F1	0	13	1	5	1	20
	F2	0	2	0	4	1	7
	Sum[*1]	0/0	15/15	1/1	9/9	2/2	27/27
HK-13	F1	11	13	2	5	0	31
	F2	9	16	1	2	1	29
	Sum[*1]	20/69	29/51	3/3	7/7	1/1	60/131
HK-14	F1	9	16	2	6	0	33
	F2	4	15	0	4	0	23
	Sum[*1]	13/63	31/40	2/2	10/10	0/0	56/115

[*1]Numbers examined/existing numbers in the colony

[*2]Dead queen, existence was inferred from the genotype analysis of workers
